# West African Policy Dialogue—impulse for better data: progress towards better analytics and better decisions

**DOI:** 10.1186/s12919-026-00362-8

**Published:** 2026-04-23

**Authors:** Gideon Kwarteng Acheampong, Fortress Yayra Aku, John Amuasi, Sylvia Annang, Silvia Argimon, Ernest Konadu Asiedu, Joseph Humphrey Kofi Bonney, Reinhard Busse, Benjamin Emikpe, Esteban Garcia-Gallo, Jonathan Mawutor Gmanyami, Anne Meierkord, Sam Kofi Newton, Bernard Nkrumah, Benjamin Nyakutsey, Daniel Opoku, Paul Elvis Onyango Ouma, Michael Owusu, Wilm Quentin

**Affiliations:** 1 German West African Centre for Global Health and Pandemic Prevention, Kumasi, Ghana; 2 Kwame Nkrumah University of Science and Technology, Kumasi, Ghana; 3 Disease Surveillance Department, Ghana Health Service, Accra, Ghana; 4 Department of Epidemiology and Biostatistics, Fred N. Binka School of Public Health, University of Health and Allied Sciences, Hohoe Campus, Hohoe, Ghana; 5 Kumasi Centre for Collaborative Research in Tropical Medicine, Kumasi, Ghana; 6 Bernhard Nocht Institute for Tropical Medicine, Hamburg, Germany; 7 Robert Koch Institute, Berlin, Germany; 8 National Centre for the Coordination of Early Warning and Response Mechanism, Accra, Ghana; 9 Noguchi Memorial Institute for Medical Research, University of Ghana, Accra, Ghana; 10 Department of Healthcare Management, Technical University, Berlin, Germany; 11 International Severe Acute Respiratory and Emerging Infection Consortium (ISARIC), Pandemic Sciences Institute, University of Oxford, Oxfordshire, UK; 12 African Field Epidemiology Network, Accra, Ghana; 13 Policy Planning, Monitoring and Evaluation Directorate, Ministry of Health, Accra, Ghana; 14 West and Central Africa Emergency Preparedness and Response Hub, WHO Regional Office for Africa, Dakar, Senegal; 15 Department of Public and Planetary Health, University of Bayreuth, Bayreuth, Germany

**Keywords:** Disease surveillance, Data analytics, Pandemic preparedness, Policy dialogue, Ghana

## Abstract

In a context of increasing efforts towards the establishment of a Regional Health Data Hub for the African Region, the 2024 West African Policy Dialogue brought together researchers and policymakers from seven West African countries in a two-day meeting in Aburi, Ghana. This report provides a high-level summary of the discussions at the meeting. The forum emphasized that the use of poor, incomplete, or inaccurate data will have negative consequences, regardless of the sophistication of the analytic tools used. New technologies have emerged that can support the generation and effective use of data. Yet, governments in West-Africa struggle to maximize the benefits of these technologies, including genomic surveillance, real-time data generation, and supranational data integration and exchange. Policies are needed that support and regulate new technologies and contribute to greater capabilities for better data.

## Introduction

The increasing frequency and complexity of emerging and re-emerging infectious diseases with pandemic potential pose critical challenges to global health security, particularly in West Africa, where surveillance, preparedness, and response systems remain vulnerable [1, 2]. Key drivers, including environmental degradation, rapid urbanisation, and intensified global connectivity, have accelerated the risk of cross-border pathogen transmission and underscored the imperative for robust pandemic prevention, preparedness, and response (PPPR) frameworks [3]. The COVID-19 pandemic further highlighted the indispensable role of timely, accurate, and integrated data as the foundation for effective PPPR and informed decision-making [4].

In this context, the West African Policy Dialogue (PD) on Pandemic Preparedness, convened biennially by the German-West African Centre for Global Health and Pandemic Prevention (G-WAC) in partnership with the World Health Organization (WHO) Hub for Pandemic and Epidemic Intelligence, the Robert Koch Institute, and the Ghana Ministry of Health, functions as a pivotal platform for multisectoral knowledge exchange. Operating under principles of neutrality and confidentiality (Chatham House Rule), the PD fosters implementation-focused discourse among policymakers, researchers, and public health practitioners to harmonise technical and policy perspectives essential for strengthening regional PPPR capacities. The 2024 PD explicitly focused on “Capabilities for Better Data, Better Analytics, Better Decisions”, underscoring the foundational role of data quality, accessibility, and interoperability in enhancing analytic capacity and evidence-informed policymaking in the region.

Despite significant progress since the COVID-19 pandemic, including the widespread adoption of digital Integrated Disease Surveillance and Response (IDSR) systems, expansion of genomic sequencing infrastructure, and improvements in event-based surveillance, challenges persist that undermine the establishment of an integrated and responsive data ecosystem in West Africa. These include continued reliance on paper-based data collection in peripheral health facilities, attrition of skilled human resources, siloed institutional arrangements across human, animal, and environmental health sectors, and insufficient interoperability among heterogeneous data platforms. The underrepresentation of veterinary and environmental health stakeholders in key policy dialogues further hinders the operationalisation of One Health approaches, limiting multisectoral collaboration that is vital for the early detection and control of zoonotic diseases.

This report synthesises discussions from the 2024 West African PD, convening participants from ministries of health, research institutions, and universities across seven West African countries- Ghana, Burkina Faso, The Gambia, Liberia, Sierra Leone, Cameroon, and Cote D’Ivoire; highlighting regional experiences and articulating priorities to enhance data generation, integration, and utilisation for pandemic preparedness. Thematic focus areas include (1) current limitations in data availability and utilisation, (2) strategic imperatives for integrating One Health surveillance, (3) the expanding role and constraints of genomic sequencing in pathogen surveillance, (4) harmonizing and integrating different data sources towards a (West) Africa health data space, and (5) the development of cross-sectoral policies and regulatory frameworks that facilitate sustainable data sharing and capacity building. These deliberations advance the agenda for establishing a Regional Health Data Hub aimed at enabling harmonised, timely, and actionable data exchange to bolster health security across West Africa.

Situating these insights within broader global health system strengthening and security frameworks, this report contributes to the evidence and policy discourse necessary to build resilient, data-driven PPPR capabilities in West Africa. It underscores the critical nexus between enhanced data infrastructure and improved public health outcomes, highlighting the imperative for sustained political commitment, cross-sectoral collaboration, and investments in human resources and technology to safeguard the region against future pandemics. The report follows the structure of the dialogue: (1) It first explores the current status of data generation, availability and use in West Africa before (2) addressing the question of how to achieve better integrated One Health surveillance systems. (3) It then focuses on the increasing role of genomic sequencing for pathogen surveillance, and (4) asks how to better harmonize and integrate different data sources. (5) Finally, the last section focuses on policies and regulations needed to strengthen capabilities for better data.

### Data Generation, availability and use in West Africa: current status and challenges

#### Multiple sources of data for disease surveillance and pandemic preparedness are available in the region

Multiple sources of data are available in the region to inform PPPR related decision-making. All countries have adopted the Integrated Disease Surveillance and Response (IDSR) strategy that establishes comprehensive public health surveillance and response systems, addressing priority diseases, conditions, and events across all levels of the health system. IDSR has undergone different iterations since adoption in 1998, with the latest version adopting a One Health approach and emphasizing e-IDSR. IDSR incorporates both indicator- and event-based surveillance approaches. There is a need to integrate both with other sources such as, genomic surveillance data, wastewater monitoring, routine statistics of the District Health Information and Management System 2 (DHIMS), health facility data of the health resources availability mapping system (HeRAMS), data of civil registration and vital statistics systems (CRVS), and administrative data on human resources, financing, infrastructure, medicines, and other supplies.

#### Completeness and timeliness of surveillance data has improved considerably

Considerable progress has been made since the COVID-19 pandemic with regard to completeness and timeliness of indicator-based IDSR reports submitted by countries since 2022. While only 10 out of 47 countries in the WHO AFRO region had submitted data in May 2022, this number increased to 35 countries (74%) by April 2023. This was supported by efforts to digitise data collection through e-IDSR systems as these systems streamline reporting and enable improved analytics. Progress has also been made with regard to event-based surveillance, which screens available information (e.g. from media reports) to detect any event that poses a potential acute risk to human health. Similarly, genomic sequencing has expanded significantly, allowing a better understanding of disease patterns. Cross-border surveillance is also being utilised to track disease movements and assess risks.

#### Paper-based data collection and human resource attrition are important challenges

Important challenges remain, including the continued reliance on paper-based systems, with many facilities still completing paper-based reports that need to be manually inputted into electronic systems at the district level. Human resource attrition is a problem for data collection in several countries, weakening data collection and management systems. A particular challenge is the collection of data at the community level, which requires better links and trust from communities as well as feedback mechanisms for community-level data contributors. This is further complicated by the wide-spread use of traditional medicine providers that are rarely integrated into community-based surveillance.

#### Systematic implementation of digital data collection tools could improve timeliness and completeness of data

Greater use of digital tools for data collection could considerably improve timeliness of available data. This could be based on simple data collection through Excel at health facilities or more sophisticated tools, such as SORMAS or REDCap or fully embracing e-IDSR and mobile data collection tools. Overall, progress is still needed to achieve greater timeliness, completeness, and integration of available data sources as well as greater collaboration across sectors. Even though the number of countries submitting IDSR reports has increased considerably as noted above, only 35% of countries had submitted complete reports on time, highlighting the need for further efforts to improve timeliness and completeness of reporting.

### Better integrated One Health surveillance systems

#### A One Health surveillance approach has important benefits

Traditional surveillance systems rely on separate data collection efforts for environmental health (e.g. impact of pollution, climate change), animal health (e.g. monitoring of livestock and wildlife), and human health. These are underpinned by separate institutional responsibilities for different sectors. However, an integrated One Health surveillance approach has important benefits, including: (i) Early detection of new or re-emerging pathogens in animals before spreading to humans, (ii) more comprehensive understanding of the development and spread of diseases, and (iii) improved and better coordinated public health responses. Recognition of the benefits of a One Health approach has led to considerable activity at the policy level and to the emergence of One Health policies and strategies in several countries, including Ghana and Cameroon.

#### Institutional fragmentation continues to hamper greater integration

Despite efforts to integrate the various arms of the surveillance systems, siloed approaches remain in many countries. Often separate data collection systems prevail for human, animal and environmental health that are related to different mandates and regulatory provisions governing the different institutions (e.g. Ministry of Health, Ministry of Food and Agriculture, and Ministry of Lands and Natural Resources). Funding and personnel allocated to the implementation of integrated approaches remain insufficient. Different cultures, power struggles, limited incentives for collaboration, and issues regarding the ownership of data contribute to inadequate collaboration between sectors. Data collection tools are rarely designed in a way that would allow transmission and integration of data.

#### Cross-sector collaboration, common data platforms, and interdisciplinary training programs are needed to support greater integration

More cross-sector collaboration among government entities, private sector stakeholders, and communities is needed to achieve better integration of data and to realise the potential of a One Health approach. This will require a reduction of regulatory fragmentation as well as more funding and dedicated personnel. The establishment of joint working groups, such as a One Health Steering Committee (in Ethiopia), or dedicated agencies could support better integration. Interdisciplinary training programmes for professionals from different fields can contribute to greater knowledge about the benefits of a One Health approach and to a shift of mindset that promotes collaboration. In addition, data sharing protocols, the establishment of interoperable systems, and/or the creation of common data platforms, enabling real-time data access for all relevant stakeholders would considerably improve the situation.

#### Future integrated data systems should include multiple data sources across sectors and countries

Data integration should not stop there. Ultimately, integrated data systems should include multiple different data sources on hazards and diseases threats, vector populations, water and air quality, as well as service utilisation and capacity data, socio-economic indicators, and reliable birth and death statistics. Furthermore, cross-country collaboration and sharing of data is important, in particular, in the case of cross-border movement of populations and animals. Yet regulatory provisions may need to be adjusted to enable transfer of data.

### Better pathogen surveillance based on genomic sequencing data

#### Genomic sequencing is a critical component of pathogen surveillance

Genomic sequencing identifies the genetic makeup of pathogens to monitor the spread and circulation of variations, such as new strains or variants, and is a critical component of pathogen surveillance. Genomic sequencing has informed public health decisions in several situations, including during the 2022 Marburg virus outbreak and the 2024 Dengue fever outbreak. Governments in several countries, including Ghana, Côte d’Ivoire, and the Gambia have considerably strengthened national infrastructure and capacities for genomic sequencing. On a policy level, Ghana has developed a National Genomic Surveillance Strategy for example. Yet, capacities vary widely across countries. The International Pathogen Surveillance Network (IPSN), an initiative launched in May 2023 by the WHO Hub for Pandemic and Epidemic Intelligence, aims to address the challenges of more equitable distribution of genomic capacity globally, optimal use of capacities in country, and advocacy for sustainable funding of pathogen genomic surveillance.

#### Multiple challenges persist with regard to technical capacity, human resource availability, and data integration

Genomic sequencing has not yet reached its full potential because of numerous interrelated challenges: Infrastructure-related challenges include unreliable electricity supply, intermittent internet connectivity, and a limited number of genomic sequencing facilities; human resource challenges include a lack of qualified lab technicians and bioinformaticians because of limited training capacity and brain drain; logistical challenges include procurement challenges for reagents and consumables as well as inadequate sample management; funding challenges exist with regard to purchase of reagents and maintenance and calibration of equipment as well as external quality assurance. Finally, genomic sequencing data sharing is currently not integrated into routine surveillance systems and data sharing across countries is burdened by technical, political and bureaucratic problems at all levels.

#### International support is available to strengthen national capacities

International partners, such as the WHO, IPSN, US Centres for Disease Control (CDC) and Robert Koch Institute support in-country processes to build and strengthen genomic sequencing capacities through e.g. training, funding, supplies, emergency procurement procedures, health system and policy analyses and political advocacy. An important focus is on workforce development, but staff retention challenges limit the effectiveness of capacity building efforts. There is a need for contractual agreements and attractive incentives to ensure trained individuals stay long enough to make an impact. Further infrastructural development, such as upgrading of laboratory equipment, provision of reliable power and internet connectivity, improved data sharing, and development of genomic sequencing laboratory operations and quality management SOPs is needed. It is important for countries but also for the African region to map national/regional capacity building priorities and clearly define roles and responsibilities of partners to avoid duplication of efforts. Collaborations should be strengthened between different sectors, such as between academia and national public health agencies but also between the human, animal and environmental sectors, to overcome silo working and thinking. Building trust among partners is crucial to achieve better data sharing, curation and analytics and there is a need for policies that facilitate genomic data sharing, regional cooperation and practical implementation of pathogen genomic surveillance.

### Harmonizing and integrating different data sources towards a (West-) African Health Data Space

#### Improved data curation and integration are key to obtaining greater insights from existing data

Lack of standardisation during data collection often hamper the creation of integrated databases. To enable integration, data curation is needed, which involves data cleaning and annotation. Large language models may support anonymization and clustering to transform unstructured data, but robust validation is essential to ensure reliability and accuracy. Further variable transformation, adjustment of heterogenous variable structures, and standardization are needed to facilitate data sharing and collaborative analysis. As an example, the International Severe Acute Respiratory and emerging Infection Consortium (ISARIC) with its African data hub in Kumasi, Ghana, supports clinical research networks with standardised protocols Case Report Forms (CRFs) and data integration and analysis tools. The approach could potentially inspire further efforts for data integration of different data sources.

#### Standardized data collection based on common protocols facilitates data integration

The WHO-ISARIC Clinical Characterisation Protocol (CCP) (Clinical Characterisation Protocol | ISARIC) has been widely implemented by ISARIC members to study various diseases, including Ebola, Zika, MERS, COVID-19, Dengue and Mpox. This protocol facilitates standardized data collection through the use of Case Report Forms (CRFs) that ensure consistency while allowing customization to suit specific outbreak contexts. To support the rapid development of standardized CRFs, ISARIC developed ARC (ISARICResearch/ARC.), a comprehensive, machine-readable library of questions. ARC covers a wide range of patient-related information and includes detailed metadata for each question, such as definitions, answer options, units, limits, data types, and skip logic. To operationalize ARC, ISARIC introduced BRIDGE (bridge.isaric.org), a web-based application designed to tailor ARC's functionality to meet the needs of specific outbreak scenarios that create the necessary files to digitally capture data in REDCap. To support data analysis, ISARIC developed VERTEX.

(https://github.com/ISARICResearch/VERTEX), a web-based application designed to facilitate evidence generation through the use of Reusable Analytical Pipelines and enable interactive visualization of curated datasets (a demo is available at https://demo.vertex.isaric.app/).

Digital data collection tools used by health workers in communities or health facilities can contribute to more standardised data collection. But use of multiple different tools by different categories of health professionals or different types of providers may create duplication of efforts (e.g. duplicate data entry) and problems of data integration. Existing tools, such as SORMAS, DHIS-2, and LHIMS (Lightwave Health Information Management System) operate with different variables and different levels of granularity, thus complicating aggregation and integration of different data sources.

#### Publicly available information from (social) media is increasingly used for event-based surveillance

The Epidemic Intelligence from Open Source (EIOS) initiative has become a cornerstone of public health surveillance in the WHO AFRO region. More than 38 countries are contributing to the EOIS-based early warning system. The system is detecting about half of all public health events in the region as the first source of information. Increasing use of AI may enable processing of more diverse data sources, including data from communities and local radio stations. However, data from the EIOS is not yet integrated into other surveillance systems, limiting use to its own web-based interface.

#### Achieving targets for the establishment of the WHO AFRO Regional Health Data Hub requires greater efforts at country level

National data harmonisation projects building on intersectoral collaborative efforts of relevant institutions and partners are needed. More work is needed to strengthen relationships between different actors, to build trust, and to create awareness of the advantages of interoperability of different data collection systems. These will be essential steps to prepare for the establishment of the Regional Health Data Hub.

### Policies and regulations to strengthen capabilities for better data

#### National policies on data collection, storage and use should reflect country and regional data needs

Many countries have national data policies and provisions for the transfer of data abroad. Where these are not (yet) available, they will need to be developed to enable establishment of the Regional Health Data Hub. When new national policies and strategies are needed, these should be based on national collaborative cross-sector and cross-institutional dialogues. It is key that national policies reflect country and regional data needs. In countries, where integrative cross-sector data collection policies are already available, problems may exist with regard to implementation. In these cases, it is equally important to convene stakeholders to develop a tailored and realistic implementation strategy.

#### Policies should be based on an integrative cross-sectoral One Health approach

Policies are needed that focus on integration of data from different sources, including civil registration and vital statistics systems, epidemiological data, clinical data, pathogen data (from animals and humans), genomic sequencing data, health service utilisation data, and data on environmental hazards. Involvement of all stakeholders in the development of such policies is key. Unfortunately, the 2024 West African PD itself was not a good example in this regard as important stakeholders from the veterinary and environmental sector were underrepresented.

#### Strengthening data collection at the community level is important and requires trust

Effective surveillance has to start at the community level. Improving surveillance at this level requires trust from the community. More efforts are needed to strengthen community level surveillance and trust in the system. This is particularly important in a context, where most people still rely on traditional medicine providers and where formal health service providers, including community level health workers may not be the first point of contact.

#### National data collection policies should prioritise digital data collection approaches

Data collection needs to become digital by default in the future to improve timeliness and completeness of data. Digital data collection could improve cost-effectiveness, but to achieve the merits of digital data collection, existing systems need to be better integrated. For example, current efforts of introducing SORMAS or other digital tools often lead to duplicate data entry and burden health workers unnecessarily. Having harmonised data collection approaches may reduce the required efforts for later data curation and integration. In addition, data should be findable, accessible, interoperable and reusable (F.A.I.R).

#### More investments are needed into training and retaining professionals

Data do not speak for itself. Sufficient numbers of highly skilled professionals are needed to translate existing data into information, and in turn to better decisions. Currently available numbers of specialised experts, e.g. in bioinformatics, are limited. In addition, a stronger focus is needed on retention of trained staff, because there is a high degree of attrition and brain drain.

#### Reaching better decisions based on data requires a stronger evidence ecosystem

The formal and informal linkages and interactions between different actors as well as their capacities and resources in the use of evidence need to be strengthened to develop a stronger evidence-ecosystem. Policy dialogues that bring together relevant stakeholders, exploring and analysing existing problems together, based on the best available evidence, may also play a role in efforts aiming to strengthen the evidence ecosystem. This is because trust of involved stakeholders is an essential resource for data integration efforts. A common understanding of existing problems and joint deliberations about the benefits and drawbacks of potential solutions can contribute to the emergence of trust in evidence-informed policy-making.

## Future outlook

Overall, discussions from the dialogue underscore that while progress has been made in enhancing the quality and diversity of data sources for disease surveillance and pandemic prevention, significant systemic challenges remain. The continued use of paper-based systems and workforce attrition impede timely data access and analysis. In genomic surveillance, persistent limitations in technical capacity, human resources, and system interoperability further constrain effectiveness. The lack of standardised protocols and inconsistencies in tools and data variables across health sectors inhibit efficient data harmonisation and integration.

To advance disease and genomic surveillance efforts, it is imperative to invest in comprehensive digital transformation strategies, including the phased replacement of paper-based systems and expansion of digital infrastructure. Workforce development programs must address attrition by building sustainable capacity in epidemiology, bioinformatics, and data science. Standardised data protocols and interoperable tools should be developed and adopted across sectors, guided by policies that promote One Health integration and support community-based surveillance initiatives. Coordinated efforts across local, national, and international levels will be critical to building resilient, data-driven health systems.

## Data Availability

The datasets supporting the conclusions of this article are included within the article.

## Abbreviations

ARC: Analysis and Research Compedium
BRIDGE: BioResearch Integrated Data Tool GEnerator
CCP: Clinical Characterisation Protocol
CDC: US Centers for Disease Control and Prevention
CRF: Case Report Form
CRVS: Civil Registration and Vital Statistics Systems
DHIMS-2: District Health Information Management System 2
e-IDSR: Electronic Integrated Disease Surveillance and Response
EIOS: Epidemic Intelligence from Open Source
FAIR: Findable, Accessible, Interoperable and Reusable
G-WAC: German West African Centre for Global Health and Pandemic Prevention
HeRAMS: Health Resources Availability Mapping System
IDSR: Integrated Disease Surveillance and Response
IPSN: International Pathogen Surveillance Network
ISARIC: International Severe Acute Respiratory and Emerging Infection Consortium
LHIMS: Lightwave Health Information Management System
PD: Policy Dialogue
PPPR: Pandemic Prevention, Preparedness, and Response
SOP: Standard Operating Procedures
SORMAS: Surveillance Outbreak Response Management and Analysis
VERTEX: Visual Evidence & Research Tool for Exploration
WHO AFRO: World Health Organization African Region
